# Supplementary figures and images for: An extracellular vesicle targeting ligand that binds to Arc proteins and facilitates Arc transport in vivo
Source: eLife. 2023 Jun 16;12:e82874. doi: 10.7554/eLife.82874 (PMC10289811; doi:10.7554/eLife.82874)

(-) control  
V5-Sas<sup>Short</sup> GOF  
V5-Sas<sup>FL</sup> GOF

L EV L EV L EV

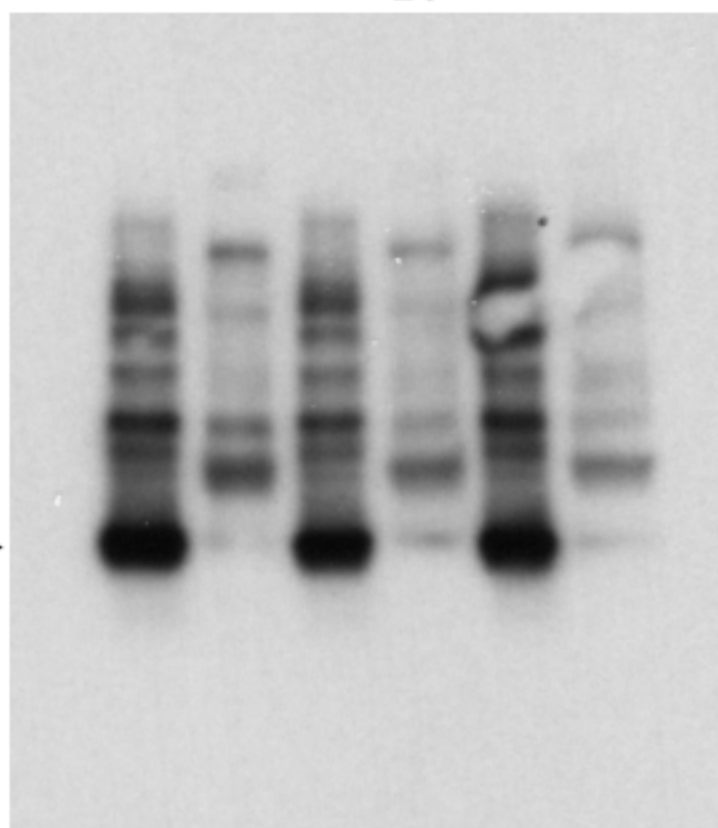

IB:  $\beta$ -Actin

42 kDa ►

Supplement: Figure 1—source data 1. [file elife-82874-fig1-data1.zip › Fig 1 Source Data/Fig 1 Full Raw Data/Fig 1c-IB-beta-Actin Labelled.pdf]

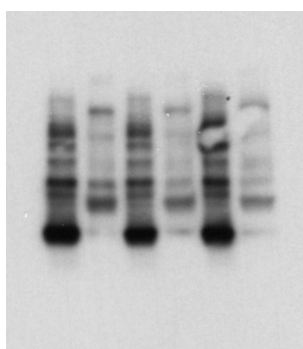

Supplement: Figure 1—source data 1. [file elife-82874-fig1-data1.zip › Fig 1 Source Data/Fig 1 Full Raw Data/Fig 1c-IB-beta-Actin.pdf]

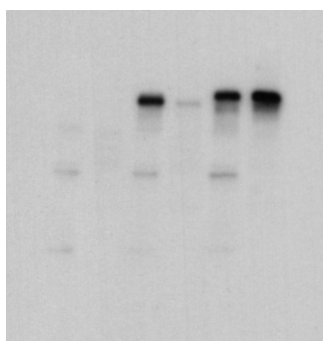

Supplement: Figure 1—source data 1. [file elife-82874-fig1-data1.zip › Fig 1 Source Data/Fig 1 Full Raw Data/Fig 1c-IB-V5.pdf]

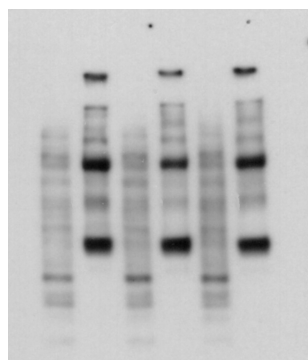

Supplement: Figure 1—source data 1. [file elife-82874-fig1-data1.zip › Fig 1 Source Data/Fig 1 Full Raw Data/Fig 1c-IB-wg.pdf]

(-) control  
V5-Sas<sup>Short</sup> GOF  
V5-Sas<sup>FL</sup> GOF

L EV L EV L EV

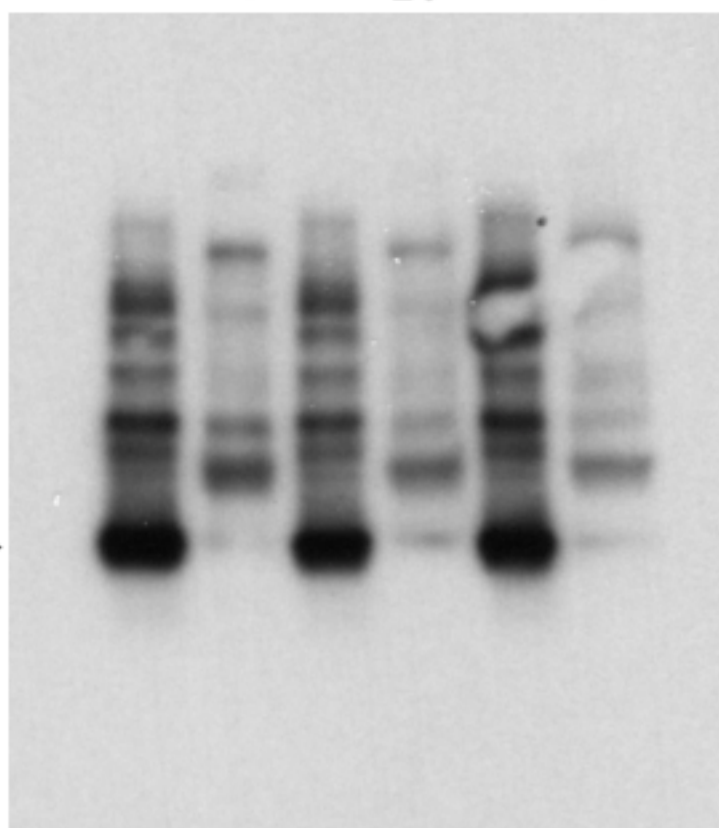

IB:  $\beta$ -Actin

42 kDa ►

Supplement: Figure 1—source data 1. [file elife-82874-fig1-data1.zip › Fig 1 Source Data/Fig 1 Labelled Raw Data/Fig 1c-IB-beta-Actin Labelled.pdf]

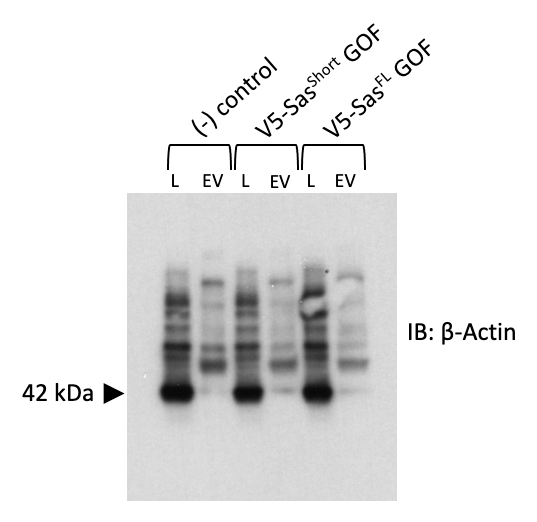

Supplement: Figure 1—source data 1. [file elife-82874-fig1-data1.zip › Fig 1 Source Data/Fig 1 Labelled Raw Data/Fig 1c-IB-beta-Actin Labelled.png]

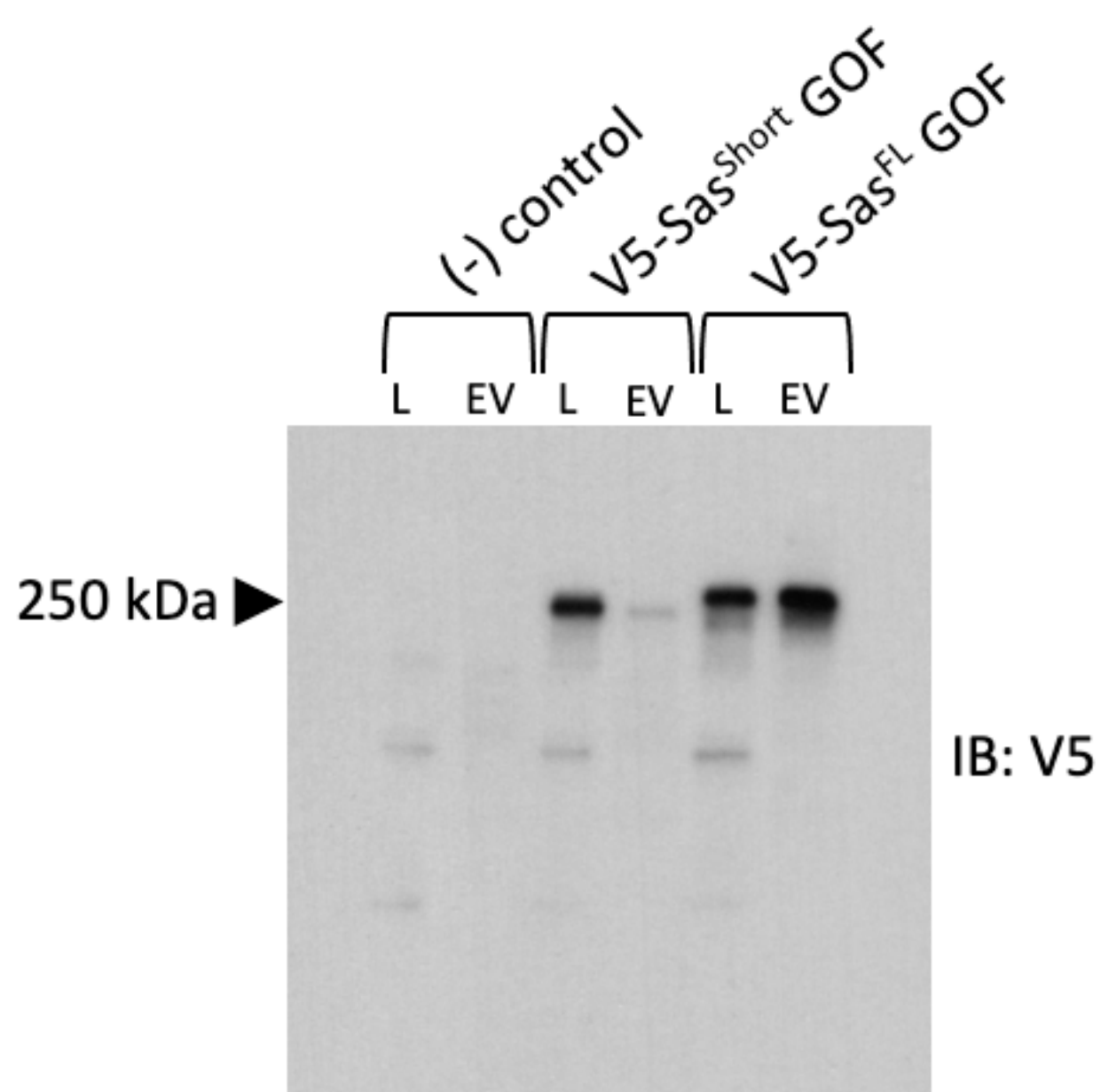

Supplement: Figure 1—source data 1. [file elife-82874-fig1-data1.zip › Fig 1 Source Data/Fig 1 Labelled Raw Data/Fig 1c-IB-V5 Labelled.pdf]

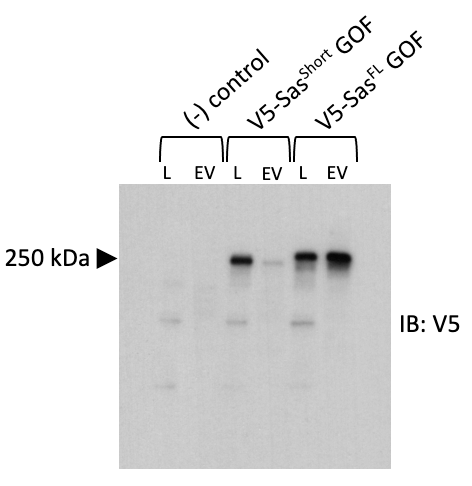

Supplement: Figure 1—source data 1. [file elife-82874-fig1-data1.zip › Fig 1 Source Data/Fig 1 Labelled Raw Data/Fig 1c-IB-V5 Labelled.png]

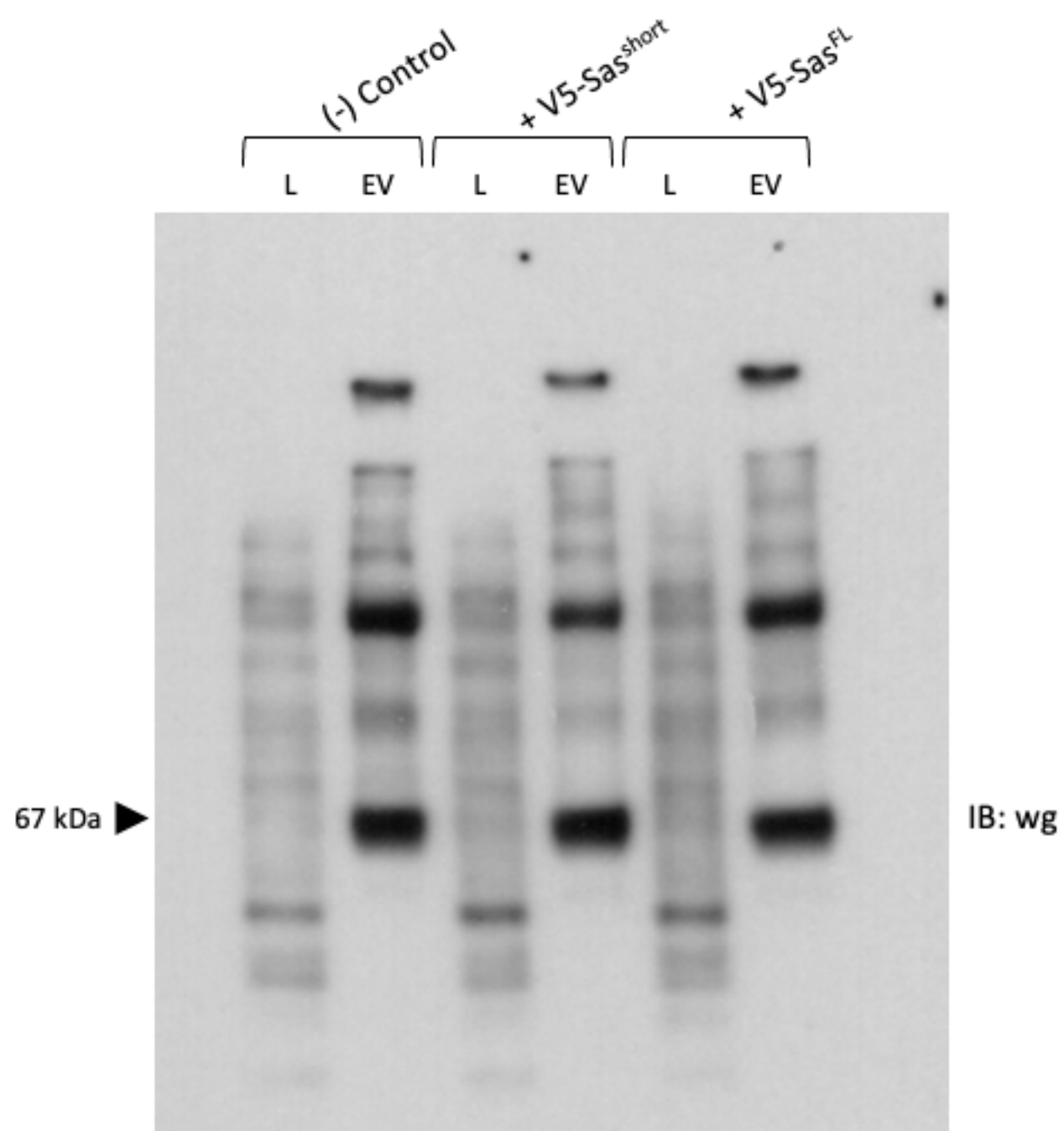

Supplement: Figure 1—source data 1. [file elife-82874-fig1-data1.zip › Fig 1 Source Data/Fig 1 Labelled Raw Data/Fig 1c-IB-wg Labelled.pdf]

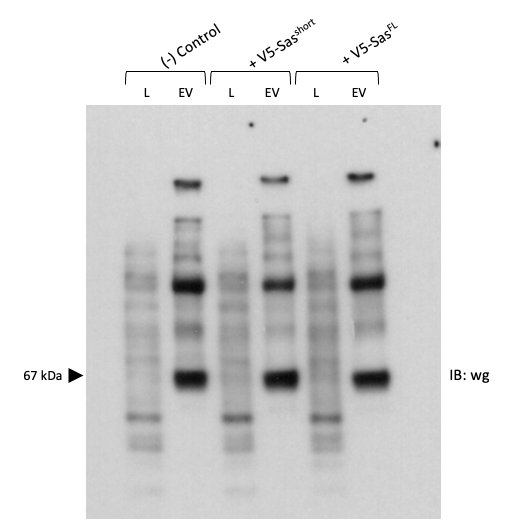

Supplement: Figure 1—source data 1. [file elife-82874-fig1-data1.zip › Fig 1 Source Data/Fig 1 Labelled Raw Data/Fig 1c-IB-wg Labelled.png]

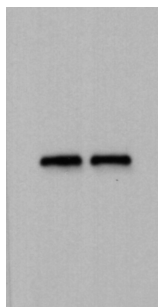

Supplement: Figure 1—figure supplement 1—source data 1. [file elife-82874-fig1-figsupp1-data1.zip › Fig 1-supp 1 Source Data/Supp Fig 1 Full Raw Data/Supp Fig 1a-IB-beta-Tub Raw Data.pdf]

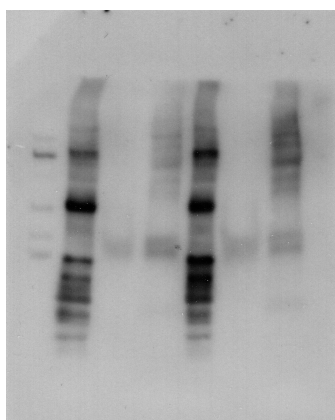

Supplement: Figure 1—figure supplement 1—source data 1. [file elife-82874-fig1-figsupp1-data1.zip › Fig 1-supp 1 Source Data/Supp Fig 1 Full Raw Data/Supp Fig 1a-IB-sas-PAPC Raw Data.pdf]

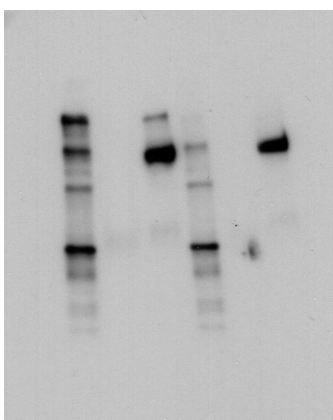

Supplement: Figure 1—figure supplement 1—source data 1. [file elife-82874-fig1-figsupp1-data1.zip › Fig 1-supp 1 Source Data/Supp Fig 1 Full Raw Data/Supp Fig 1a-IB-sas-PBPD.pdf]

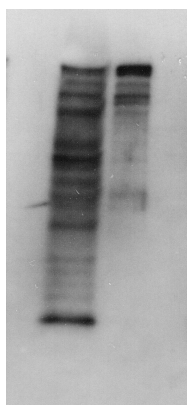

Supplement: Figure 1—figure supplement 1—source data 1. [file elife-82874-fig1-figsupp1-data1.zip › Fig 1-supp 1 Source Data/Supp Fig 1 Full Raw Data/Supp Fig 1e-1 IB-V5 Raw data.pdf]

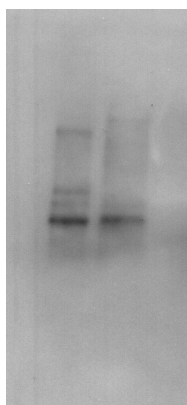

Supplement: Figure 1—figure supplement 1—source data 1. [file elife-82874-fig1-figsupp1-data1.zip › Fig 1-supp 1 Source Data/Supp Fig 1 Full Raw Data/Supp Fig 1e-2 IB-wg Raw data.pdf]

S2/Sas<sup>FL</sup>

S2/Sas<sup>short</sup>

50 kDa ►

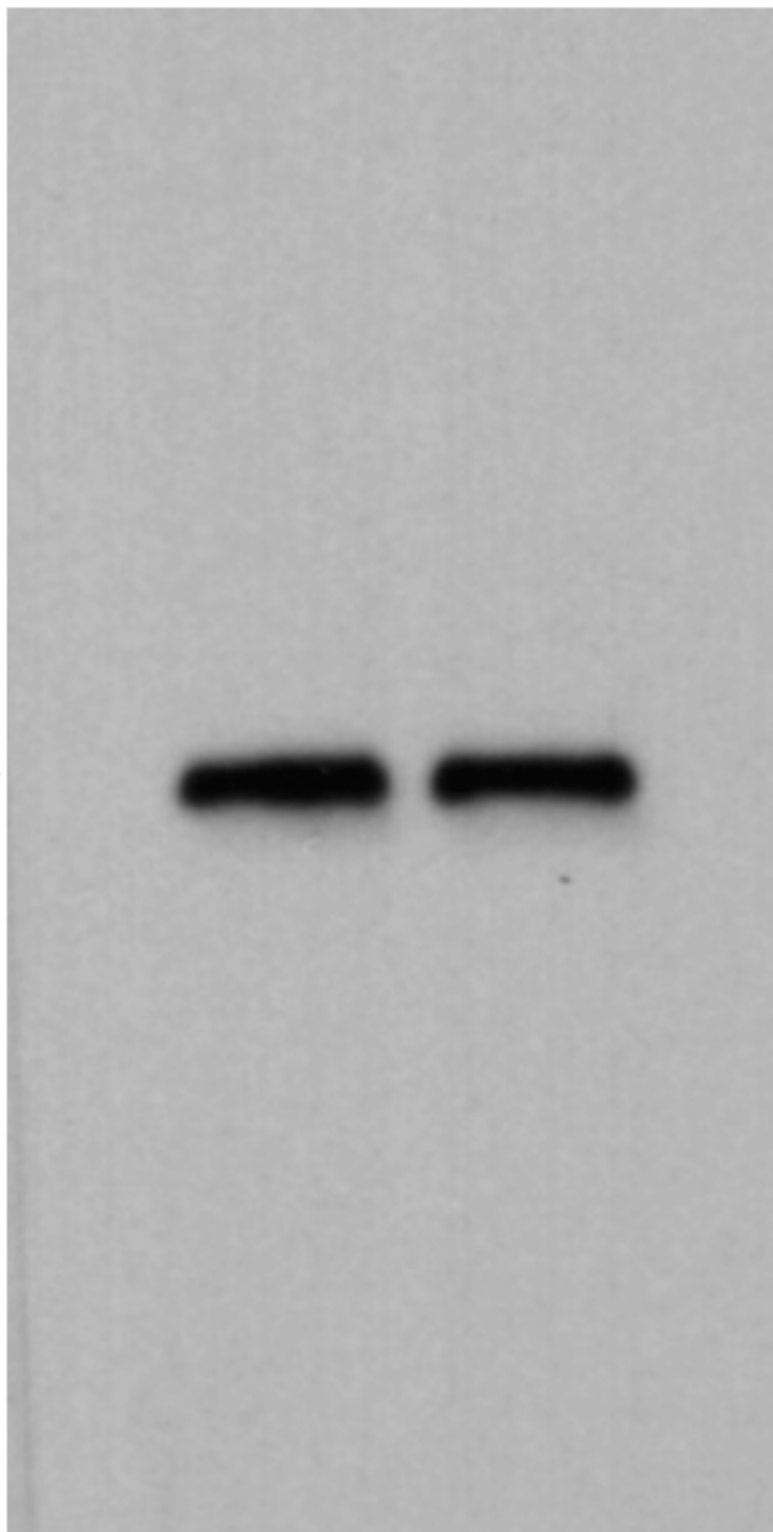

IB: β-Tubulin

Supplement: Figure 1—figure supplement 1—source data 1. [file elife-82874-fig1-figsupp1-data1.zip › Fig 1-supp 1 Source Data/Supp Fig 1a Labelled Raw Data/Supp Fig 1a-IB-beta-Tubulin Labelled Raw Data.pdf]

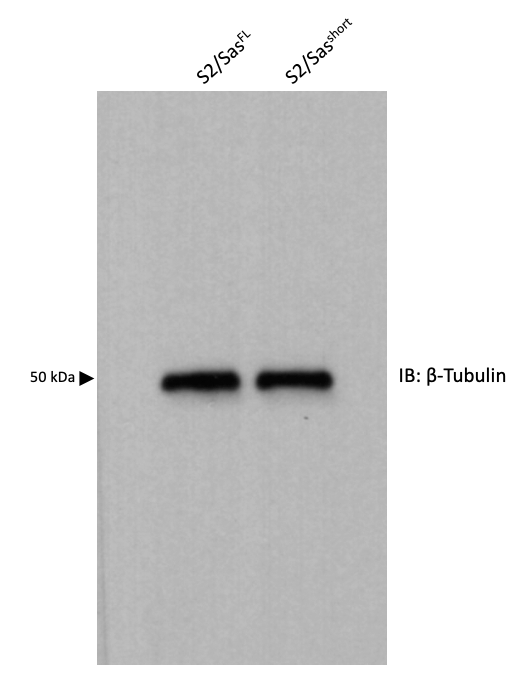

Supplement: Figure 1—figure supplement 1—source data 1. [file elife-82874-fig1-figsupp1-data1.zip › Fig 1-supp 1 Source Data/Supp Fig 1a Labelled Raw Data/Supp Fig 1a-IB-beta-Tubulin Labelled Raw Data.png]

IB: sas-PA/PC

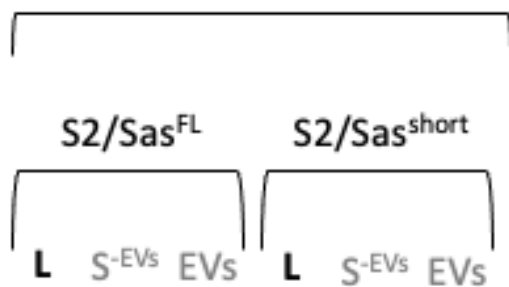

IB: sas-PB/PD

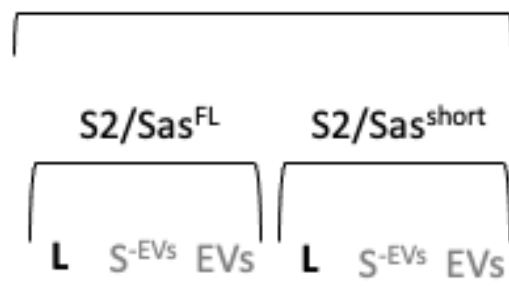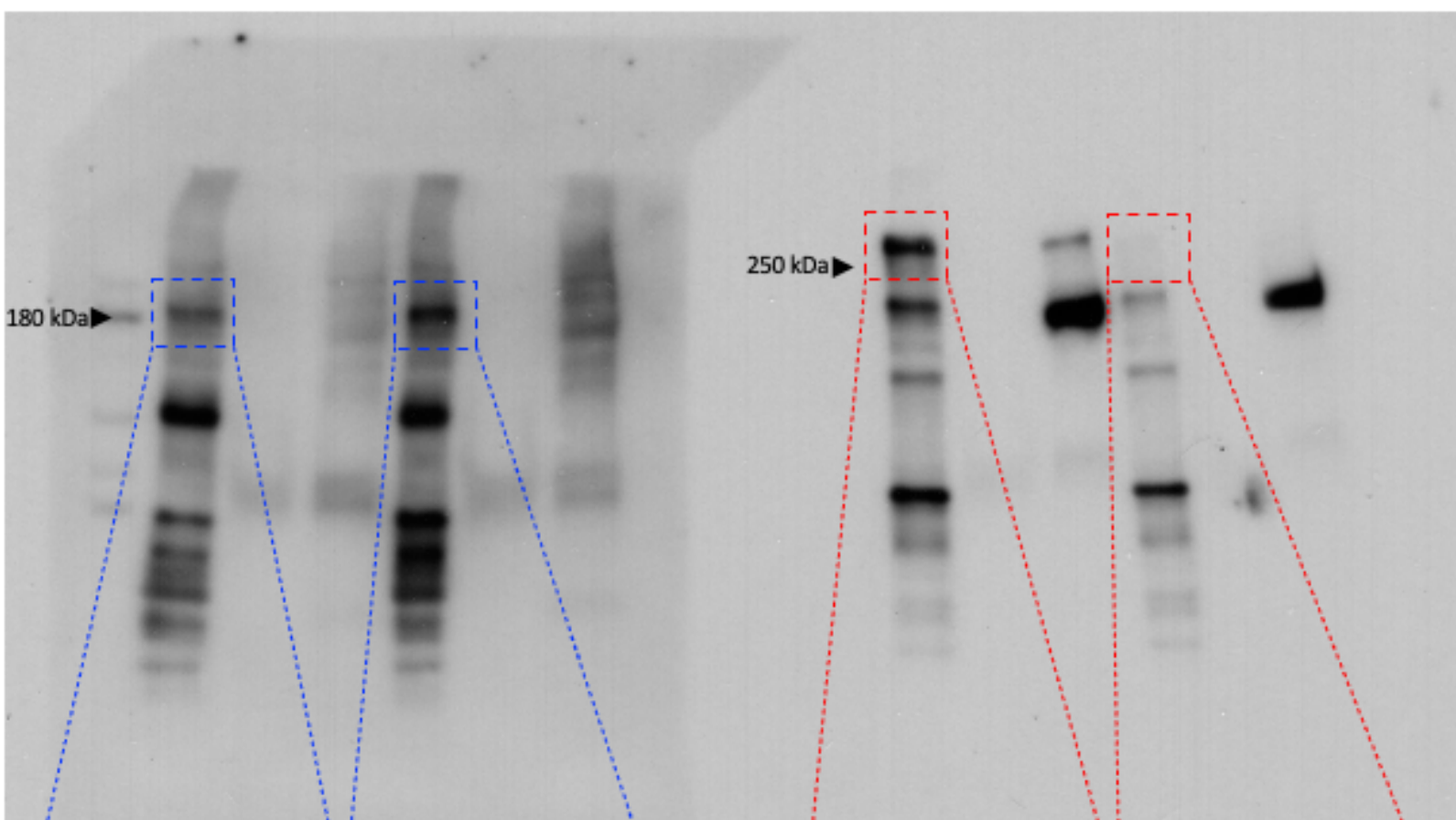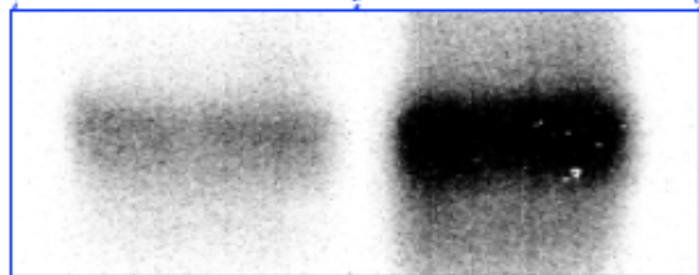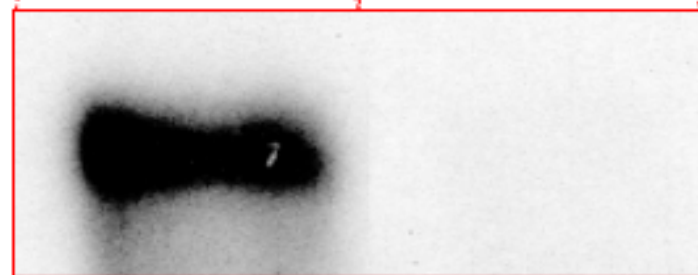

L: Lysate  
S-EVs: Supernatant w/o EVs  
EVs: Extracellular Vesicles

Supplement: Figure 1—figure supplement 1—source data 1. [file elife-82874-fig1-figsupp1-data1.zip › Fig 1-supp 1 Source Data/Supp Fig 1a Labelled Raw Data/Supp Fig 1a-IB-sas-PA,PC & PB,PD Labelled Raw Data.pdf]

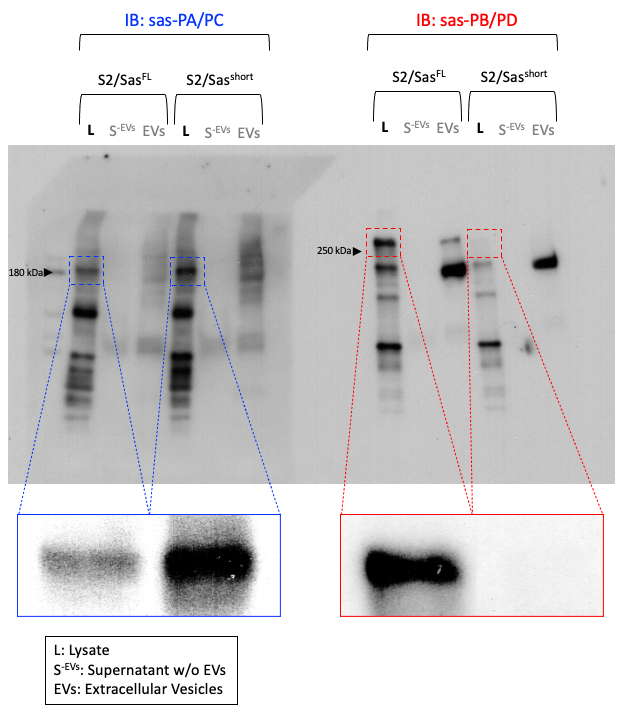

Supplement: Figure 1—figure supplement 1—source data 1. [file elife-82874-fig1-figsupp1-data1.zip › Fig 1-supp 1 Source Data/Supp Fig 1a Labelled Raw Data/Supp Fig 1a-IB-sas-PA,PC & PB,PD Labelled Raw Data.png]

EVs from S2/Sas<sup>FL</sup>

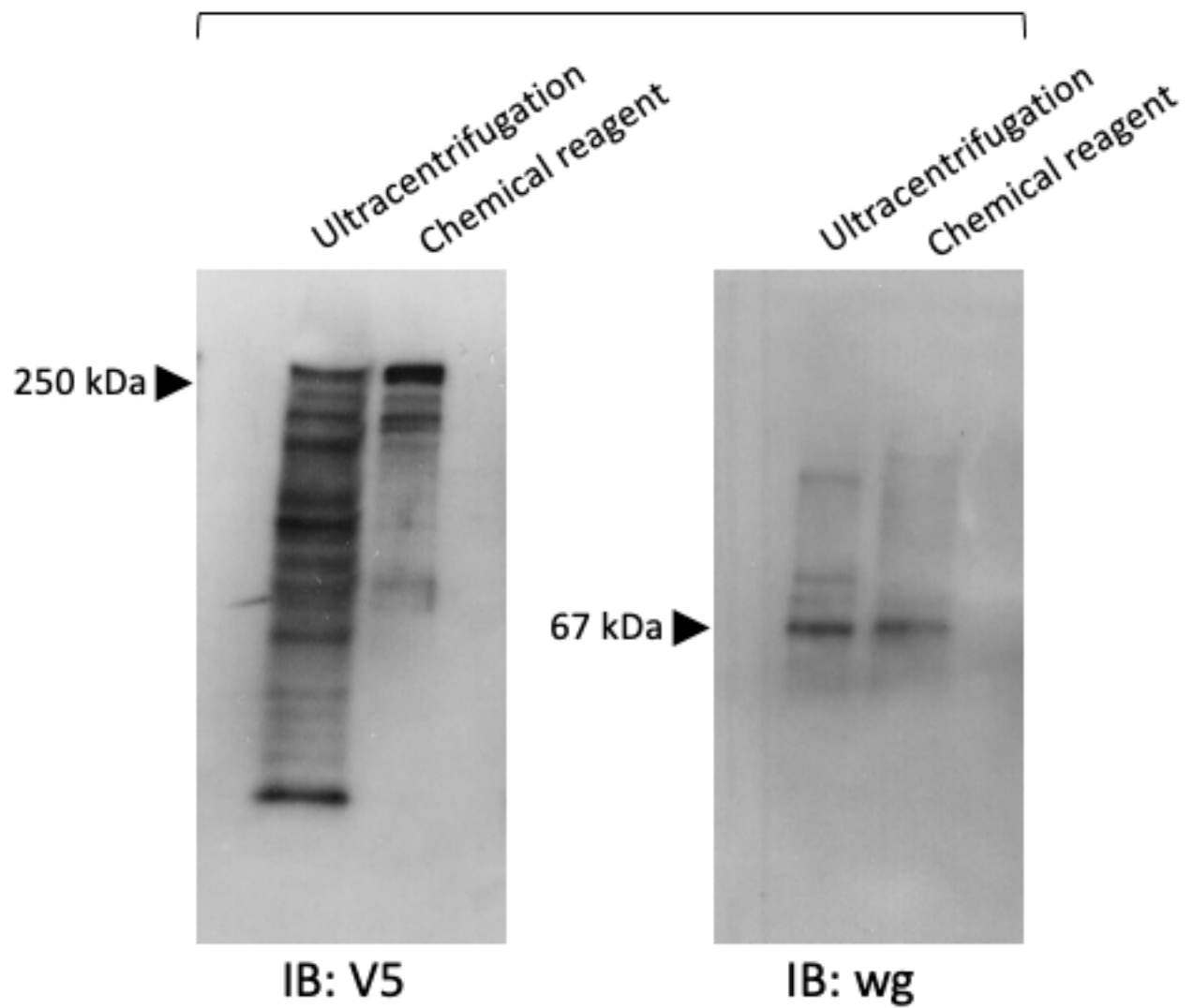

Supplement: Figure 1—figure supplement 1—source data 1. [file elife-82874-fig1-figsupp1-data1.zip › Fig 1-supp 1 Source Data/Supp Fig 1a Labelled Raw Data/Supp Fig 1e-IB-V5 & wg Labelled Raw Data.pdf]

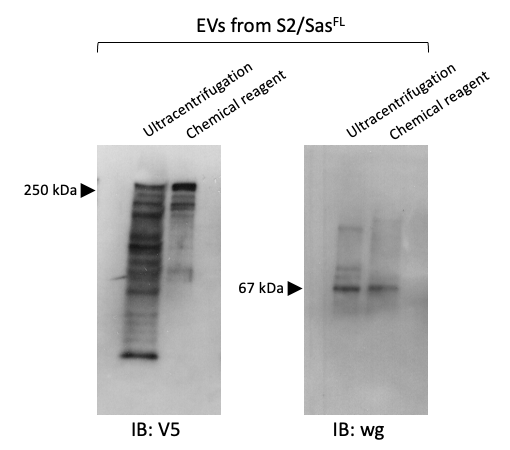

Supplement: Figure 1—figure supplement 1—source data 1. [file elife-82874-fig1-figsupp1-data1.zip › Fig 1-supp 1 Source Data/Supp Fig 1a Labelled Raw Data/Supp Fig 1e-IB-V5 & wg Labelled Raw Data.png]

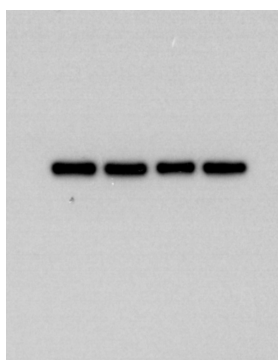

Supplement: Figure 3—source data 1. [file elife-82874-fig3-data1.zip › Fig 3-Source Data/Full Raw Data/Fig 3G anti-Tub Raw Data.pdf]

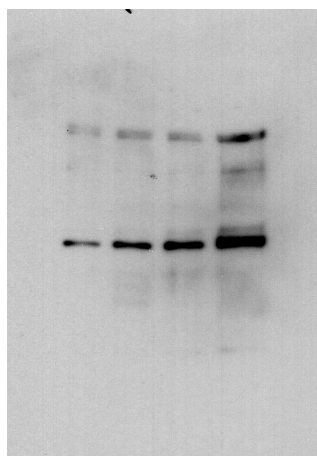

Supplement: Figure 3—source data 1. [file elife-82874-fig3-data1.zip › Fig 3-Source Data/Full Raw Data/Fig 3G anti-V5 Raw data.pdf]

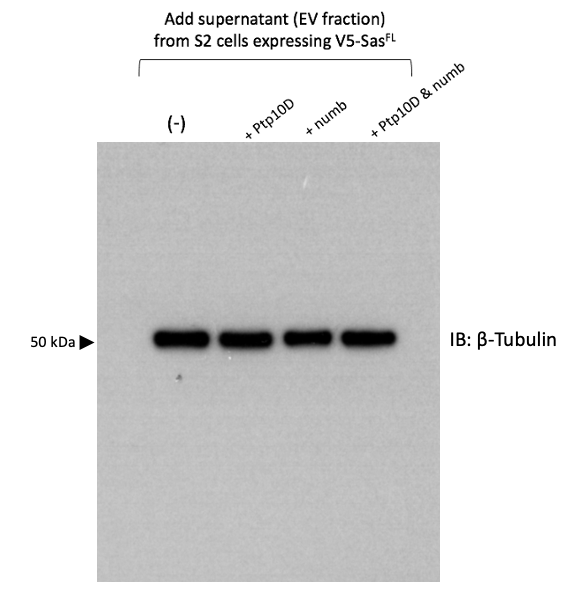

Supplement: Figure 3—source data 1. [file elife-82874-fig3-data1.zip › Fig 3-Source Data/Labelled Raw Data/Fig 3F IB-beta Tubulin Labelled.png]

Add supernatant (EV fraction)  
from S2 cells expressing V5-Sas<sup>FL</sup>

(-)

+ Ptp10D

+ numb

+ Ptp10D & numb

250 kDa

IB: V5

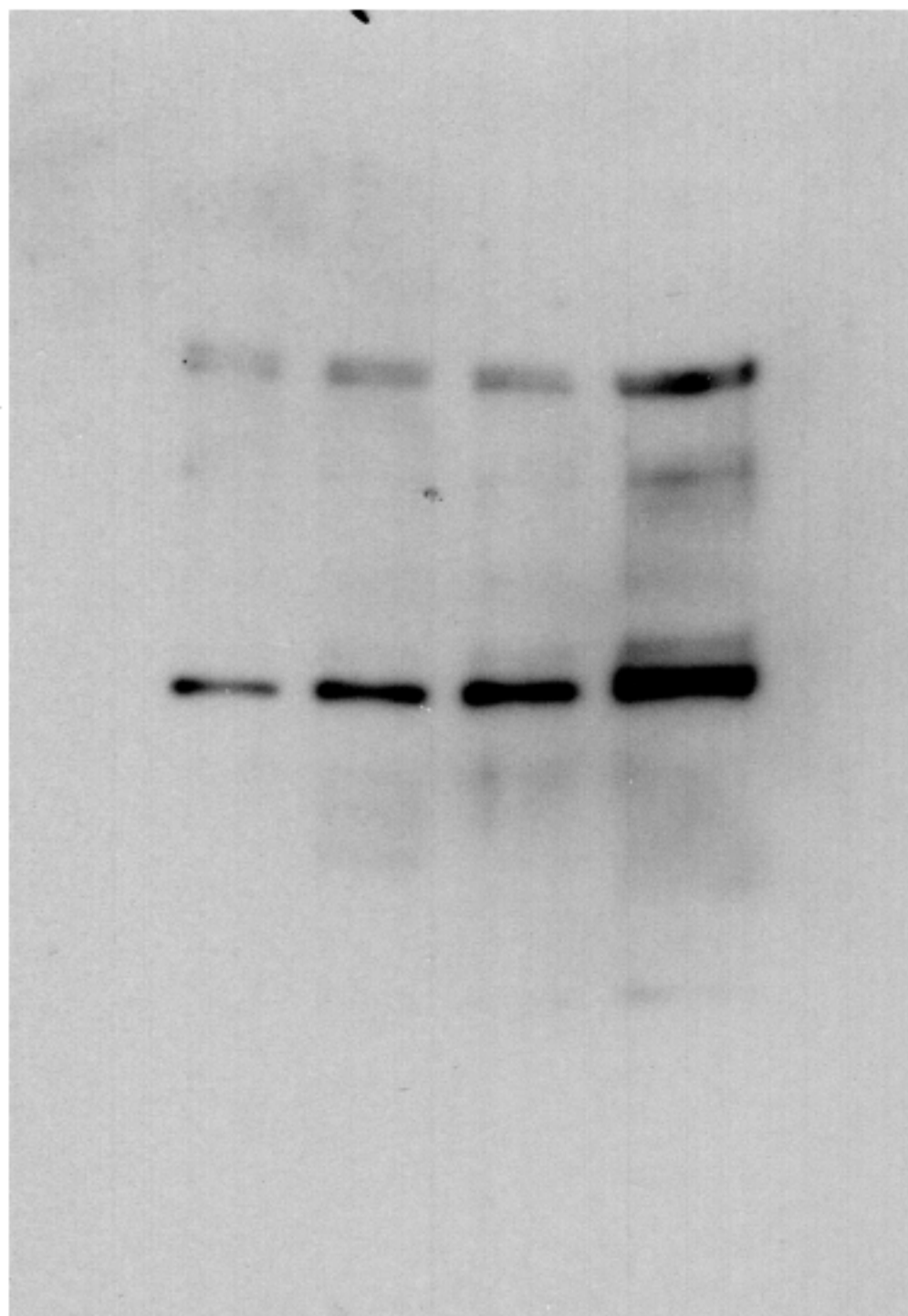

Supplement: Figure 3—source data 1. [file elife-82874-fig3-data1.zip › Fig 3-Source Data/Labelled Raw Data/Fig 3F IB-V5 Labelled.pdf]

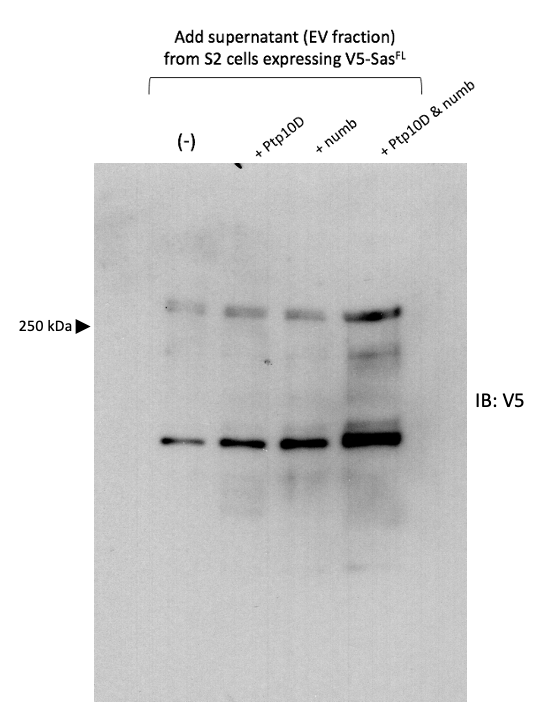

Supplement: Figure 3—source data 1. [file elife-82874-fig3-data1.zip › Fig 3-Source Data/Labelled Raw Data/Fig 3F IB-V5 Labelled.png]

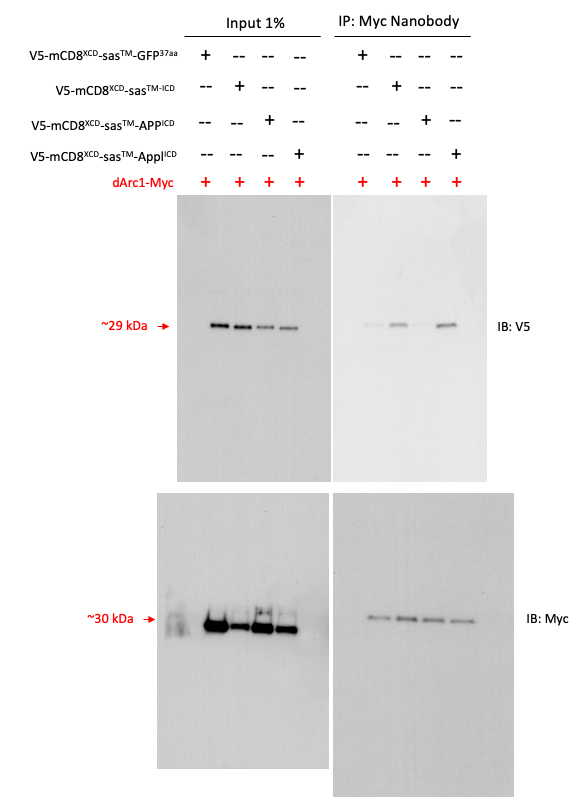

Supplement: Figure 4—source data 1. [file elife-82874-fig4-data1.zip › Fig 4C Labelled Raw Data/Fig 4C dArc1-Myc Labelled.png]

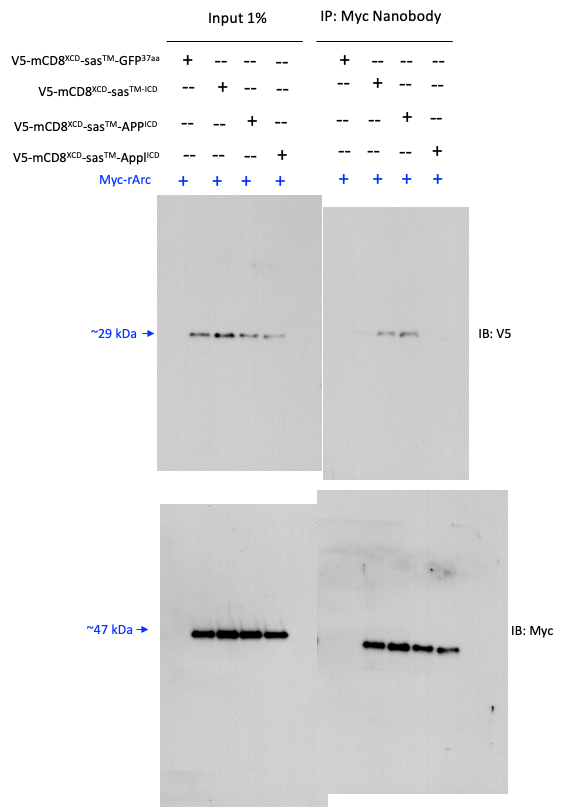

Supplement: Figure 4—source data 1. [file elife-82874-fig4-data1.zip › Fig 4C Labelled Raw Data/Fig 4C rArc-Myc Labelled.png]

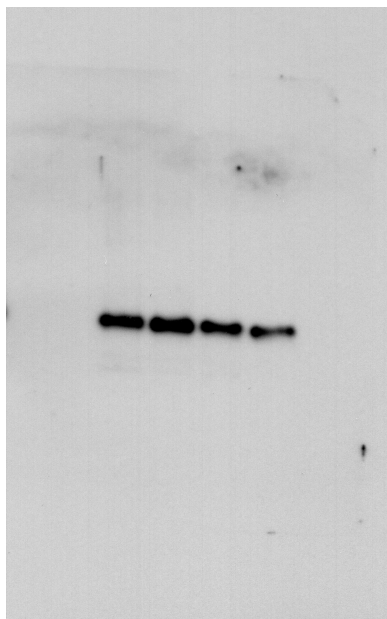

Supplement: Figure 4—source data 2. [file elife-82874-fig4-data2.zip › Fig 4C #8 IP IB-Myc Raw data.pdf]

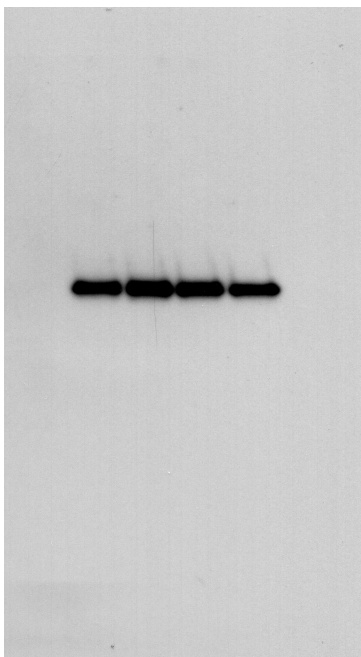

Supplement: Figure 4—source data 2. [file elife-82874-fig4-data2.zip › Fig 4C #7 Input IB-Myc Raw data.pdf]

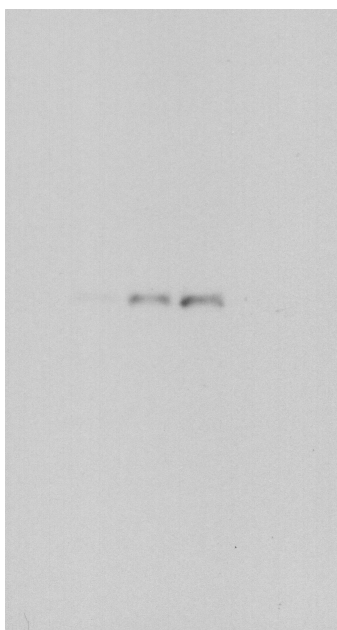

Supplement: Figure 4—source data 2. [file elife-82874-fig4-data2.zip › Fig 4C #6 IP IB-V5 Raw data.pdf]

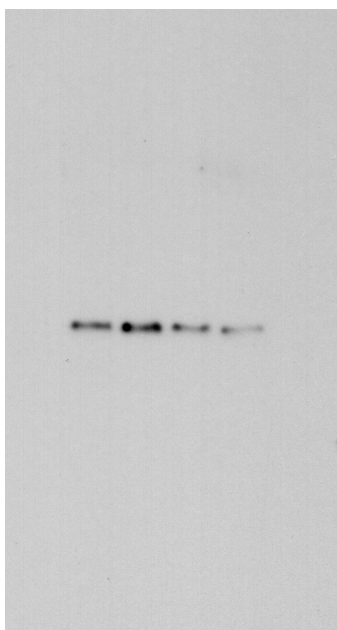

Supplement: Figure 4—source data 2. [file elife-82874-fig4-data2.zip › Fig 4C #5 Input IB-V5 Raw data.pdf]

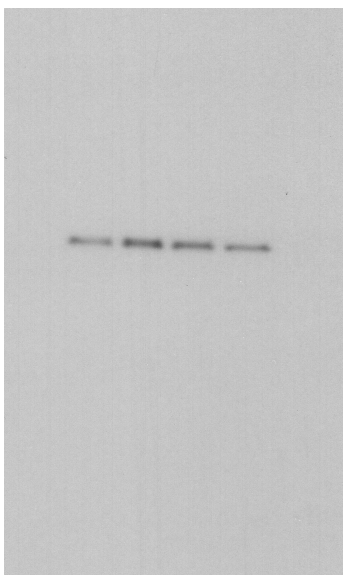

Supplement: Figure 4—source data 2. [file elife-82874-fig4-data2.zip › Fig 4C #4 IP IB-Myc Raw data.pdf]

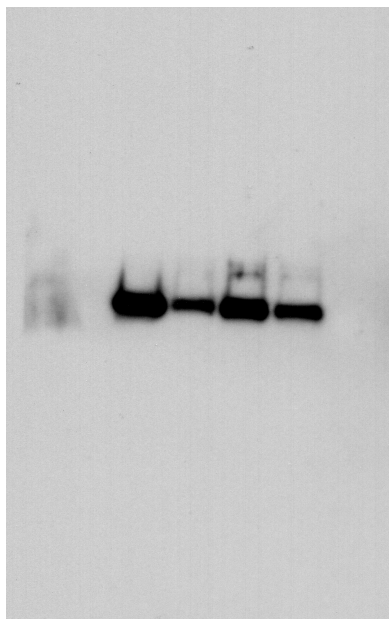

Supplement: Figure 4—source data 2. [file elife-82874-fig4-data2.zip › Fig 4C #3 Input IB-Myc Raw data.pdf]

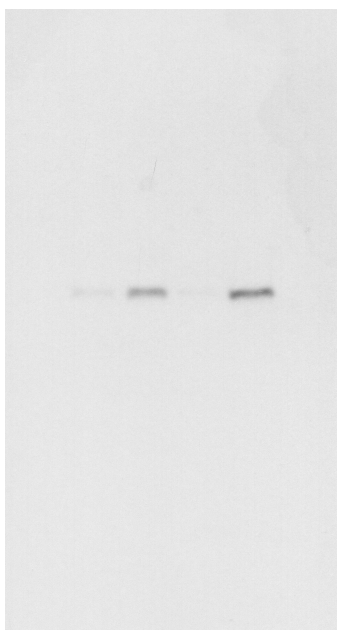

Supplement: Figure 4—source data 2. [file elife-82874-fig4-data2.zip › Fig 4C #2 IP IB-V5 Raw data.pdf]

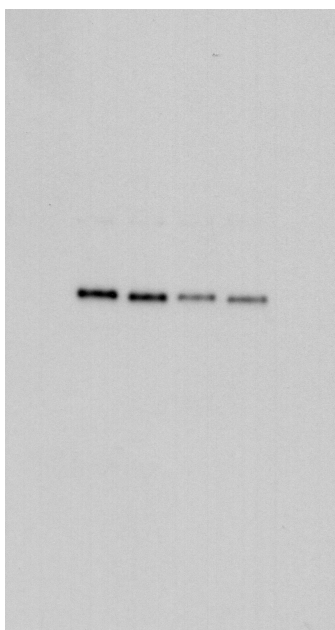

Supplement: Figure 4—source data 2. [file elife-82874-fig4-data2.zip › Fig 4C #1 Input IB-V5 Raw data.pdf]

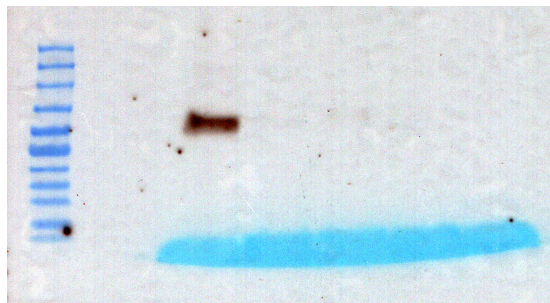

Supplement: Figure 4—source data 3. [file elife-82874-fig4-data3.zip › Fig 4D Full Raw Data/Fig 4D1-IB-GST.pdf]

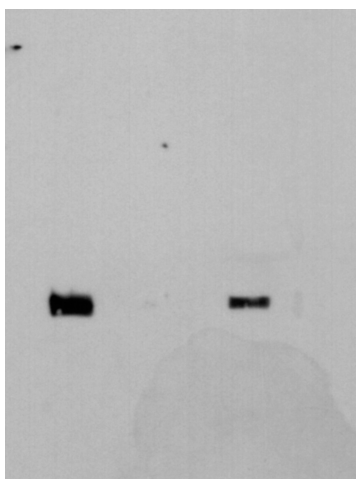

Supplement: Figure 4—source data 3. [file elife-82874-fig4-data3.zip › Fig 4D Full Raw Data/Fig 4D2-IB-GST .pdf]

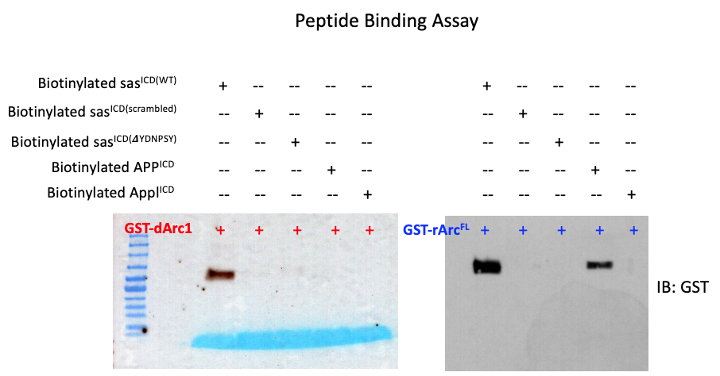

Supplement: Figure 4—source data 3. [file elife-82874-fig4-data3.zip › Fig 4D Labelled Raw Data/Fig 4D-IB-anti GST Labelled.png]

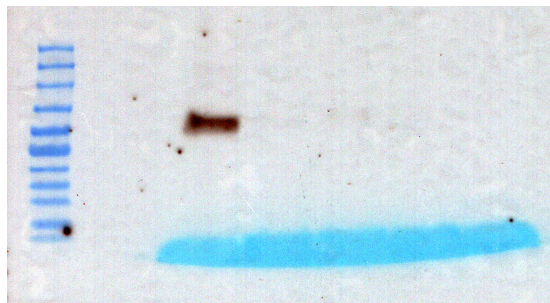

Supplement: Figure 4—source data 3. [file elife-82874-fig4-data3.zip › Fig 4D Labelled Raw Data/Fig 4D1-IB-GST.pdf]
