# Supplementary material for: An extracellular vesicle targeting ligand that binds to Arc proteins and facilitates Arc transport in vivo
Source: eLife. 2023 Jun 16;12:e82874. doi: 10.7554/eLife.82874 (PMC10289811; doi:10.7554/eLife.82874)
Supplement: Figure 3—source data 1. [file elife-82874-fig3-data1.zip › Fig 3-Source Data/Labelled Raw Data/Fig 3F IB-beta Tubulin Labelled.pdf]

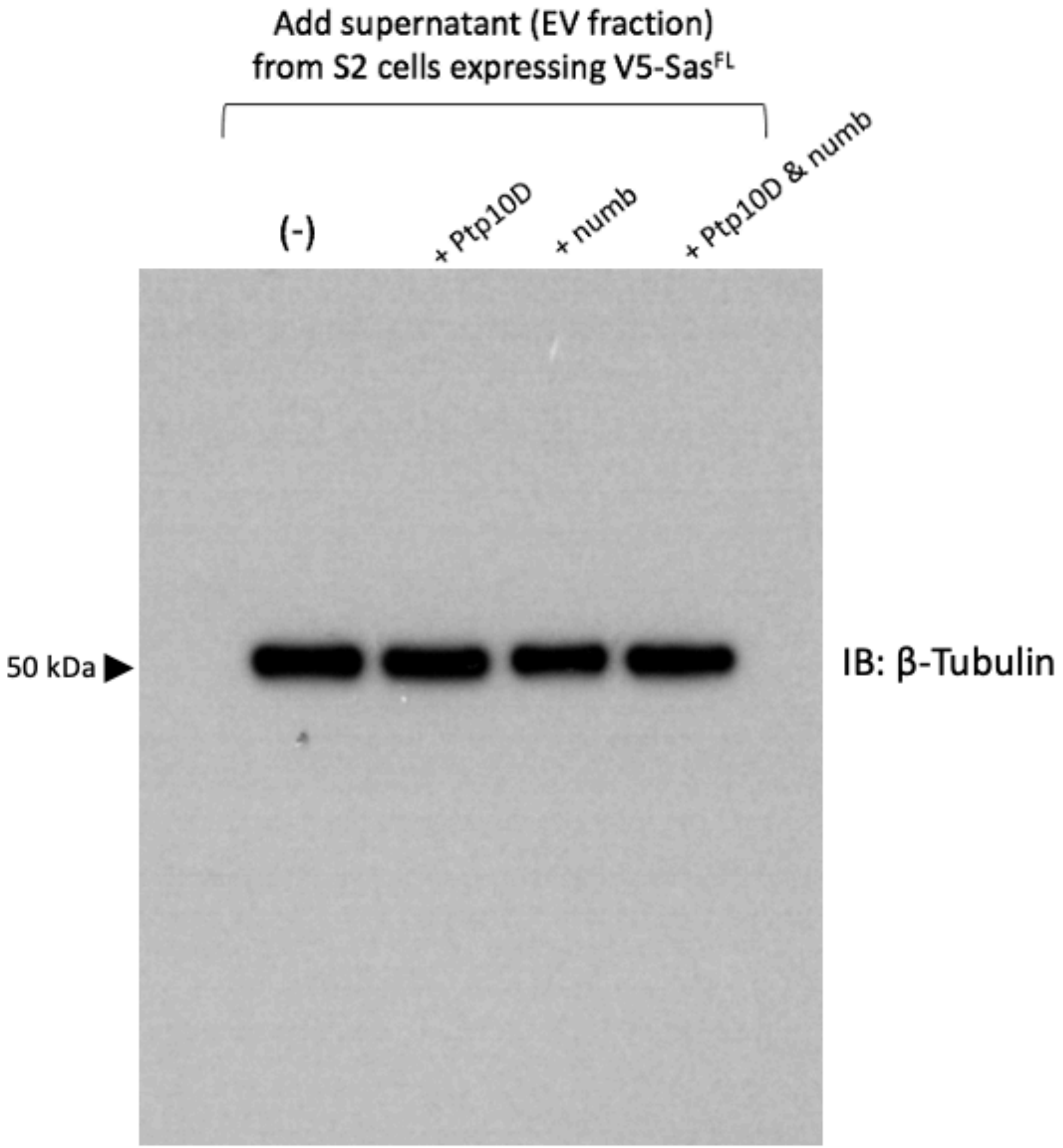
Add supernatant (EV fraction)
from S2 cells expressing V5-Sas^FL
(-)
+ Ptp10D
+ numb
+ Ptp10D & numb
50 kDa
IB: β-Tubulin
