# Supplementary material for: An extracellular vesicle targeting ligand that binds to Arc proteins and facilitates Arc transport in vivo
Source: eLife. 2023 Jun 16;12:e82874. doi: 10.7554/eLife.82874 (PMC10289811; doi:10.7554/eLife.82874)
Supplement: Figure 4—source data 1. [file elife-82874-fig4-data1.zip › Fig 4C Labelled Raw Data/Fig 4C rArc-Myc Labelled.pdf]

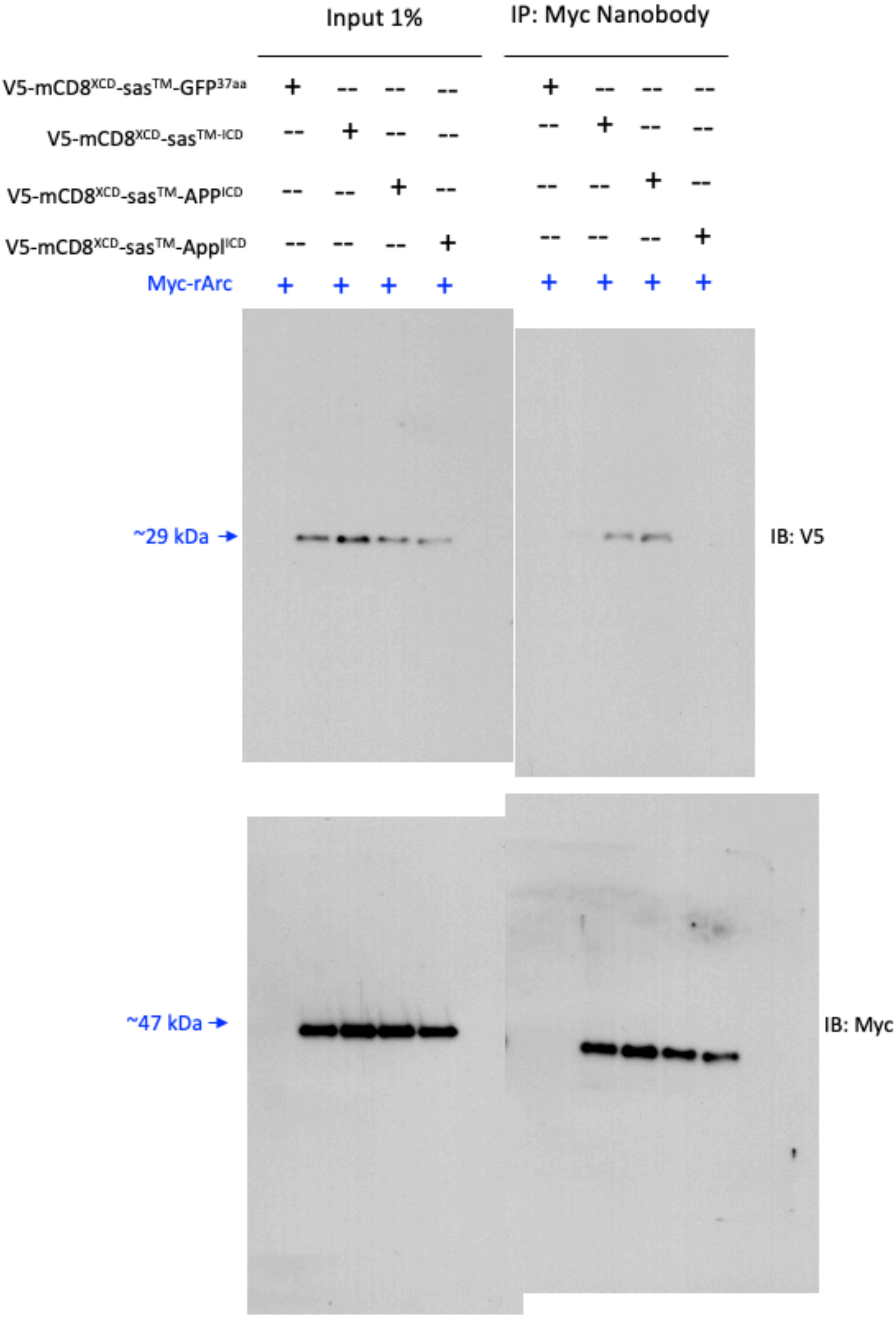
Input 1%
IP: Myc Nanobody
V5-mCD8XCD-sasTM-GFP37aa
V5-mCD8XCD-sasTM-ICD
V5-mCD8XCD-sasTM-APPICD
V5-mCD8XCD-sasTM-ApplICD
Myc-rArc
~29 kDa
IB: V5
~47 kDa
IB: Myc
