# Supplementary material for: An extracellular vesicle targeting ligand that binds to Arc proteins and facilitates Arc transport in vivo
Source: eLife. 2023 Jun 16;12:e82874. doi: 10.7554/eLife.82874 (PMC10289811; doi:10.7554/eLife.82874)

Peptide Binding Assay

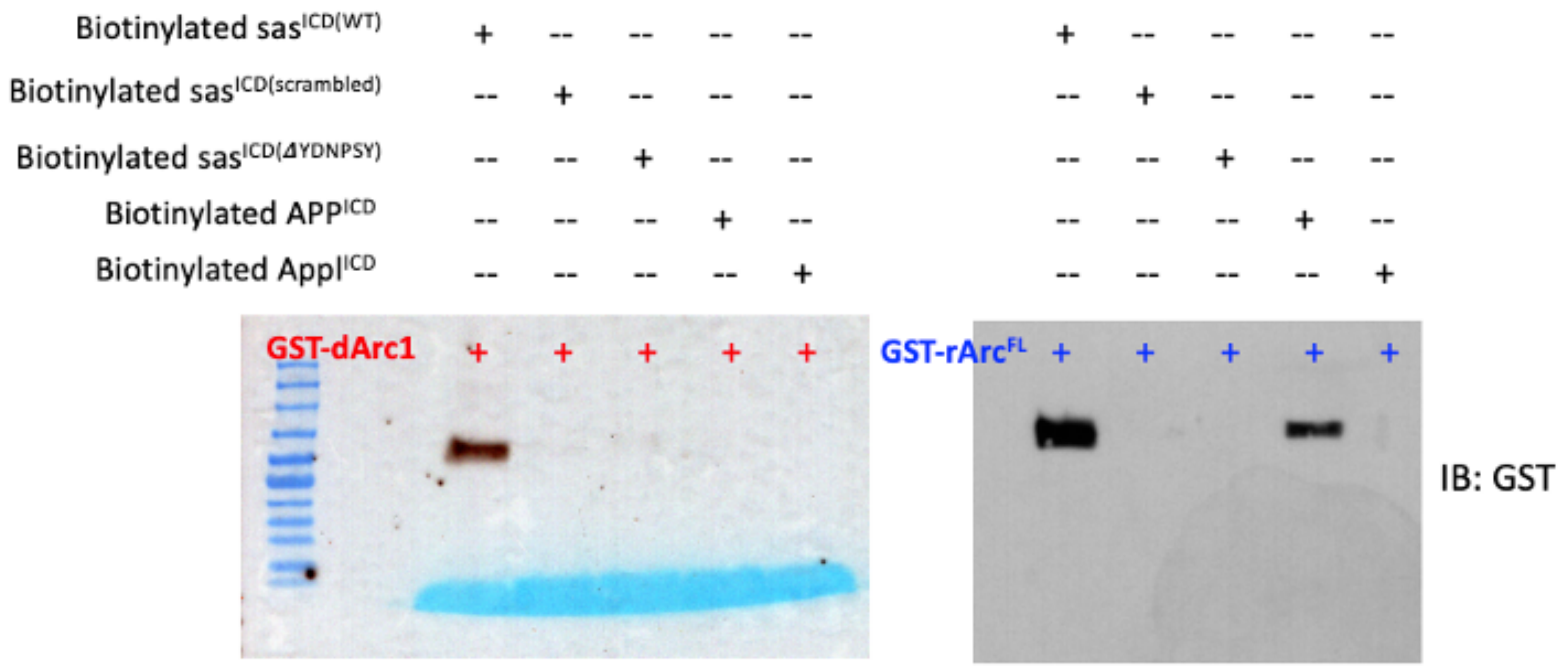

Supplement: Figure 4—source data 3. [file elife-82874-fig4-data3.zip › Fig 4D Labelled Raw Data/Fig 4D-IB-anti GST Labelled.pdf]
